# Bladder rupture after pembrolizumab immunotherapy for bladder cancer: a case report

**DOI:** 10.11604/pamj.2022.42.98.33911

**Published:** 2022-06-07

**Authors:** Ahmed Badheeb, Omar Alkhanbashi, Slah Rakrouki, Tahir Mahmood, Mashhoor Alqannas, Mohamed Badheeb, Faisal Ahmed

**Affiliations:** 1 Oncology Center, King Khalid Hospital, Najran, Saudi Arabia,; 2 Department of Urology, King Khalid Hospital, Najran, Saudi Arabia,; 3 Department of General Surgery, King Khalid Hospital, Najran, Saudi Arabia,; 4 Department of Internal Medicine, King Khalid Hospital, Najran, Saudi Arabia,; 5 Urology Research Center, Al-Thora General Hospital, Department of Urology, School of Medicine, Ibb University of Medical Science, Ibb, Yemen

**Keywords:** Perforation, bladder cancer, pembrolizumab, case report

## Abstract

Pembrolizumab is a promising checkpoint inhibitor for advanced urothelial carcinoma. Like other immunotherapies, it can cause rare immune-related adverse events. The spontaneous rupture of the urinary bladder after the intravenous injection of pembrolizumab is rare and has not been reported. Here, we present a 74-year-old man patient case of locally advanced transitional cell carcinoma of the urinary bladder who presented severe abdominal pain the same day of the second dose of pembrolizumab administration. The exploratory laparotomy revealed intraperitoneal rupture of the urinary bladder associated with peritonitis. After surgical repair, the patient's condition improved. The purpose of this report is to discuss the possible association of bladder perforation in bladder cancer with pembrolizumab immunotherapy, its management, and the importance of early recognition to prevent more fatal complications.

## Introduction

Injuries to the urinary bladder are rare and life-threatening events. Prompt diagnosis followed by surgical intervention is the key to a successful outcome [1]. The actual incidence of spontaneous rupture of the urinary bladder is uncertain; it is estimated to represent one in 126000 hospital admissions [2]. Pembrolizumab, a programmed cell death-1 checkpoint inhibitor, has high efficacy, low toxicity, and prolonged overall survival in platinum-refractory metastatic urothelial carcinoma [3]. It was approved for non-muscle-invasive bladder cancer (NMIBC) with carcinoma in situ patients who are not eligible or who have chosen not to undergo cystectomy [4]. Many cutaneous, endocrinological, and neurological side effects have been reported using pembrolizumab; in addition, gastrointestinal involvement was observed in the form of hepatitis, pancreatitis, intestinal perforation, and colitis [5]. Although infrequent, renal-related side effects associated with pembrolizumab, including; acute tubulointerstitial nephritis, nonbacterial cystitis, and nephritis [6,7]. To our knowledge, this is the first reported case of spontaneous intraperitoneal urinary bladder perforation associated with peritonitis after pembrolizumab immunotherapy in a patient with transitional cell carcinoma (TCC) of the urinary bladder NMIBC.

## Patient and observation

**Patient information:** a 74-year-old man with advanced TCC of the urinary bladder on immunotherapy with pembrolizumab presented to our emergency department with severe diffuse abdominal pain after administering the second dose of pembrolizumab. The patient was a known case of NMIBC with carcinoma in situ, who Bacillus Calmette-Guérin-unresponsive, elected not to undergo cystectomy. The patient decided to try pembrolizumab. He also a case of hypertension and diabetes mellitus on oral medication.

**Clinical findings:** regarding physical examination, the pulse rate was 105 beats per minute, the respiratory rate was 20 per minute, blood pressure was 90/60 mm/Hg, and the oral temperature was 38°C. The patient had diffuse abdominal tenderness, guarding, and detectable pelvic mass up to the umbilicus.

**Figure 1:**
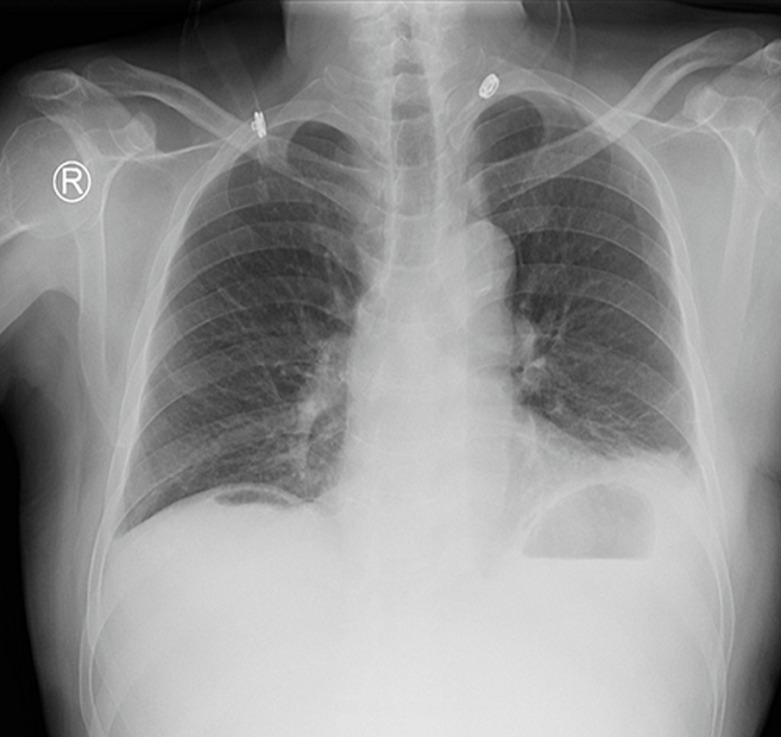
chest X-ray showing air under the diaphragm

**Diagnostic assessment:** blood tests revealed a total white blood cell count: 12 x 10^3^ ml with moderate leukocytosis and hemoglobin: 14.4 g/dl, blood urea nitrogen: 14 mg/dl, creatinine: 1.5 mg/dl. The chest radiograph demonstrated air under the diaphragm (Figure 1). The patient was diagnosed with acute abdomen and peritonitis. For that, the patient was prepared emergently for surgical operation without ultrasonography or computed tomography (CT) scan investigations.

**Figure 2:**
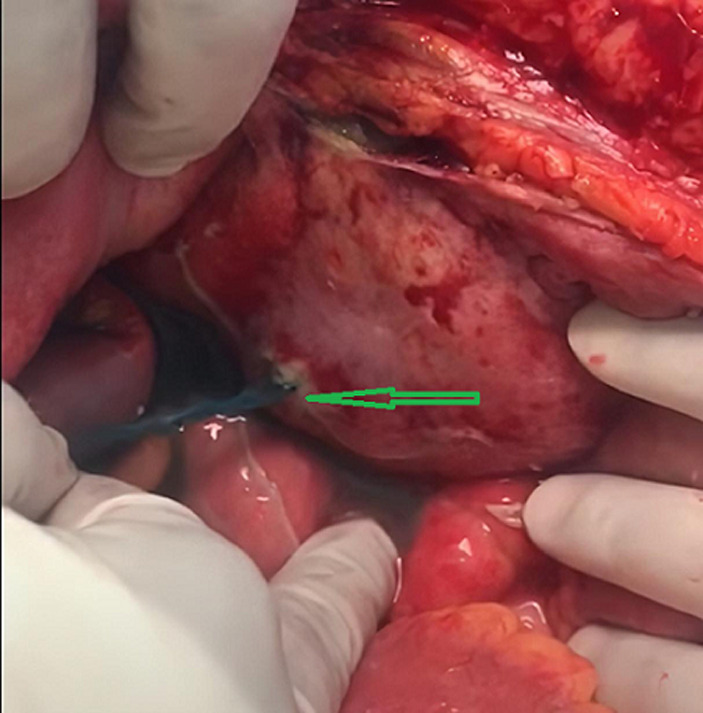
intraoperative photo showing perforation of the urinary bladder (arrow)

**Therapeutic interventions:** the patient underwent an immediate exploratory laparotomy, and it was discovered that he had a frozen pelvis, an adherent bladder to the anterior abdominal wall. The methylene blue dye test showed a 2 cm intraperitoneal perforation and another approximately 4 cm extraperitoneal perforation with urine leakage (Figure 2). The gastrointestinal tract was intact and wash-out was performed for the bladder for some necrotic tissue observed intraoperatively. Due to the unstable patient's condition during surgery, the intraperitoneal perforation was closed with a vicryl suture. The edges of the extraperitoneal perforation and the posterior abdominal wall were invaded by the tumor, difficult to recognize. In addition, a 36 French catheter scale was inserted as a drain in the retropubic space, and the Foley catheter was kept inflated in place. The patient was kept on bilateral nephrostomy drain to complete diversion of the urine post-operatively.

**Follow-up and outcome:** at postoperative day 5, the patient was discharged home with Foley’s catheter and bilateral nephrostomy drain.

**Patient perspective:** the patient said: before entering the hospital and due to the severe abdominal pain, I thought it was the end of life and that I was inevitably dead, but thanks to God and the doctors, the doctors were able to save my life. The patient thanked the doctors for all the care and medical advice they gave him.

**Written informed consent:** written informed consent was obtained from the patient for participation in our study.

## Discussion

To our knowledge, this is the first reported case of urinary bladder perforation during pembrolizumab immunotherapy from a patient with NMIBC. The possible pathogenesis of bladder rupture in bladder cancer is the precipitation of perforation on the weakened body wall by the tumor. However, the most frequent location for intraperitoneal perforation was the bladder's dome or posterior wall [1]. Spontaneous bladder rupture is more common in men with squamous cell carcinoma, as their bladder is more distensible and subsequently more prone to perforation, especially in the dome or posterior wall [8,9].

High clinical suspension is crucial for the timely diagnosis, and radiological confirmation should be established by abdominal CT and cystogram [1,10]. Our patient presented with symptoms of peritonitis and no time for additional radiologic investigation. For that, we decide on surgical exploration depending on our physical exam and plain radiographic image. Organ perforation in the setting of immunotherapy is a rare but life-threatening complication and should be considered in cancer patients receiving immunotherapy who present with acute abdomen. Its incidence rate in previous studies was 12% [1]. Colitis-induced colon perforation and fatal pneumonitis are rare but serious adverse events [11,12]. Like in our case, the main presenting symptoms are mainly those of peritonitis and, in some cases, may lead to false alarm of acute renal insufficiency, which is mainly due to the peritoneal absorption of urine [1,9].

The prognosis for spontaneous bladder rupture due to carcinoma is poor. In a previous review, most patients died within months, ranging from 10 days to 8 months. The mortality rate can range from 25% to 80%, depending on the time of diagnosis [8]. Our patient was diagnosed and operated in the same day of presentation. After confirmation of diagnosis, extraperitoneal rupture is usually treated conservatively, while intraperitoneal rupture should be treated surgically, such as primary closure, partial cystectomy, or radical cystectomy. However, a significant proportion of cases are unfit and managed conservatively using an indwelling urethral catheter and/or with bilateral nephrostomy [9,13]. Partial cystectomy was impossible in our case as the tumor was occupying most of the bladder, leaving no normal mucosa. For that, our case underwent primary repair of intraperitoneal rupture, internal Foley insertion, and bilateral nephrostomy insertion. Although no pathological specimen was submitted for study at the time of perforation, which could support our hypothesis with the presence of increased lymphocytic infiltration/inflammation; or evidence of clinical response with necrosis increasing the risk of rupture, we hypothesize that immunotherapy given with a weakened bladder wall played a significant role in the perforation.

## Conclusion

Urinary bladder perforation in the setting of pembrolizumab immunotherapy for bladder cancer should be considered among the differential diagnosis of acute abdomen and peritonitis. This case report opens the door for further confirmative studies as a hypothesis-generating.
